# Long Segment Spinal Epidural Abscess With Acute Intestinal Obstruction: A Case Report and Literature Review

**DOI:** 10.1002/ccr3.71058

**Published:** 2025-09-28

**Authors:** Shitashma Bohara, Susmin Karki, Bikas Thapa, Niharika Khanal, Bikal Ghimire, Sandeep Bohara, Amit Pradhanang, Mohan R. Sharma

**Affiliations:** ^1^ Star Hospital Lalitpur Nepal; ^2^ Maharajgunj Medical Campus Tribhuvan University Institute of Medicine Maharajgunj Nepal; ^3^ Department of Neurosurgery Tribhuvan University Teaching Hospital, Institute of Medicine Kathmandu Nepal; ^4^ Department of Gastroenterology Tribhuvan University Teaching Hospital, Institute of Medicine Kathmandu Nepal

**Keywords:** case report, epidural space, intestinal obstruction, Pott's spine, spinal epidural abscess

## Abstract

Spinal epidural abscess (SEA) is a life‐threatening condition that often presents with non‐specific symptoms, resulting in a delay in diagnosis and treatment. Rarely, it can co‐exist with acute abdominal conditions, which can further complicate the diagnosis and management. Delay in diagnosis and the presence of comorbidities may result in unfavorable outcomes. We present a case of a 55‐year‐old male diagnosed with a spinal epidural abscess presenting as low back pain, fever, and paraparesis. This case report aims to investigate the clinical presentation, diagnostic challenges, and treatment approaches for spinal epidural abscesses in low and middle‐income countries.

**Summary**

- SEA with acute intestinal obstruction is rare and diagnostically challenging; delayed recognition in comorbid patients often leads to poor outcomes.

## 1 Introduction

Spinal epidural abscess (SEA), a suppurative condition common in developing countries or in places where there is a high prevalence of substance abuse, often presents with non‐specific symptoms such as back pain, fever, or neurological deficits that can potentially lead to mis‐ or a delayed diagnosis and treatment [1, 2, 3]. Earlier studies have reported the incidence to be between 0.2 and 1.2 cases per 10,000 admissions, with an increasing trend in the recent decades [1, 2]. Risk factors include immunocompromised states such as diabetes mellitus, HIV infection, etc., spinal surgeries, or a potential local or systemic infection such as osteomyelitis, sepsis, intravenous drug use, etc. [2, 3]. Gadolinium‐enhanced MRI is the gold standard for the diagnosis [4, 5]. The coexistence of acute intestinal obstruction with a long‐segment spinal SEA is rare. Management of this patient was unique because of the urgent nature of both conditions, though the outcome was eventually negative. This report aims to inform readers of this rare co‐existence and describe the treatment strategy. This case has been reported in line with the SCARE guidelines [6].

## 2 Case History/Examination

**FIGURE 1:**
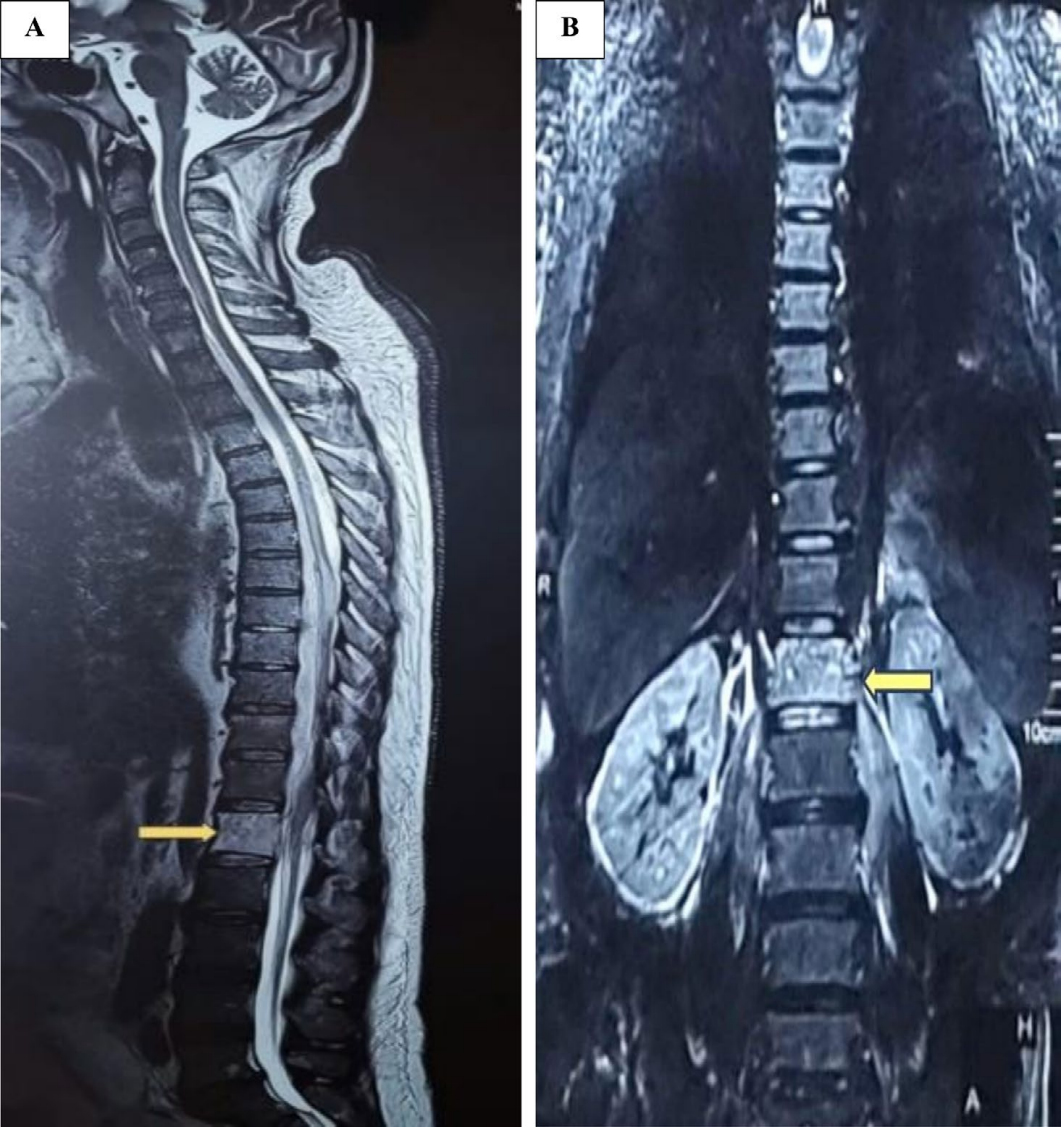
Magnetic resonance imaging T2 weighted sagittal (A) and coronal (B) sections of dorsolumbar spine showing T2 hyperintense lesion of L1 vertebra. No intracanalicular lesion was identified.

Our patient was a 55‐year‐old male from the Terai region of Nepal who developed low back pain, insidious in onset, for 1 month, with an increase in severity for 3–4 days. There was no associated limb numbness, weakness, or sphincter dysfunction. The patient was admitted locally and treated with analgesics and bed rest. However, he started to develop a fever with chills. He was noted to have a high erythrocyte sedimentation rate (ESR) of 50 mm/h (0–15 mm/h) and C‐reactive protein (CRP) of 310 mg/L (< 30 mg/L) on initial lab tests. His total leucocyte count level was revealed to be 16,300 cells/mm^3^ (4000–11,000 cells/mm^3^). His random blood sugar level had risen to 431 mg/dL (70–140 mg/dL). A gadolinium‐enhanced dorsolumbar spine MRI revealed T1 hypointense and T2/STIR hyperintensity involving most of the L1 vertebral body, suggesting infective/inflammatory pathology (Figure 1).

## 3 Differential Diagnosis, Investigations and Treatment

The patient's geographical location, clinical presentation (chronic low back pain with systemic features, including fever with chills), markedly elevated inflammatory markers (ESR and CRP), and leukocytosis were noted. Additionally, a classical imaging pattern was suggestive of spinal tuberculosis; hence, a provisional diagnosis of Pott's spine was made, and empiric anti‐tubercular therapy (ATT) was initiated while awaiting further diagnostic confirmation. On the 12th day of admission, the patient developed paraparesis and decreased sensation associated with progressive constipation and multiple episodes of vomiting. The patient was then referred to our center for further management.

**FIGURE 2:**
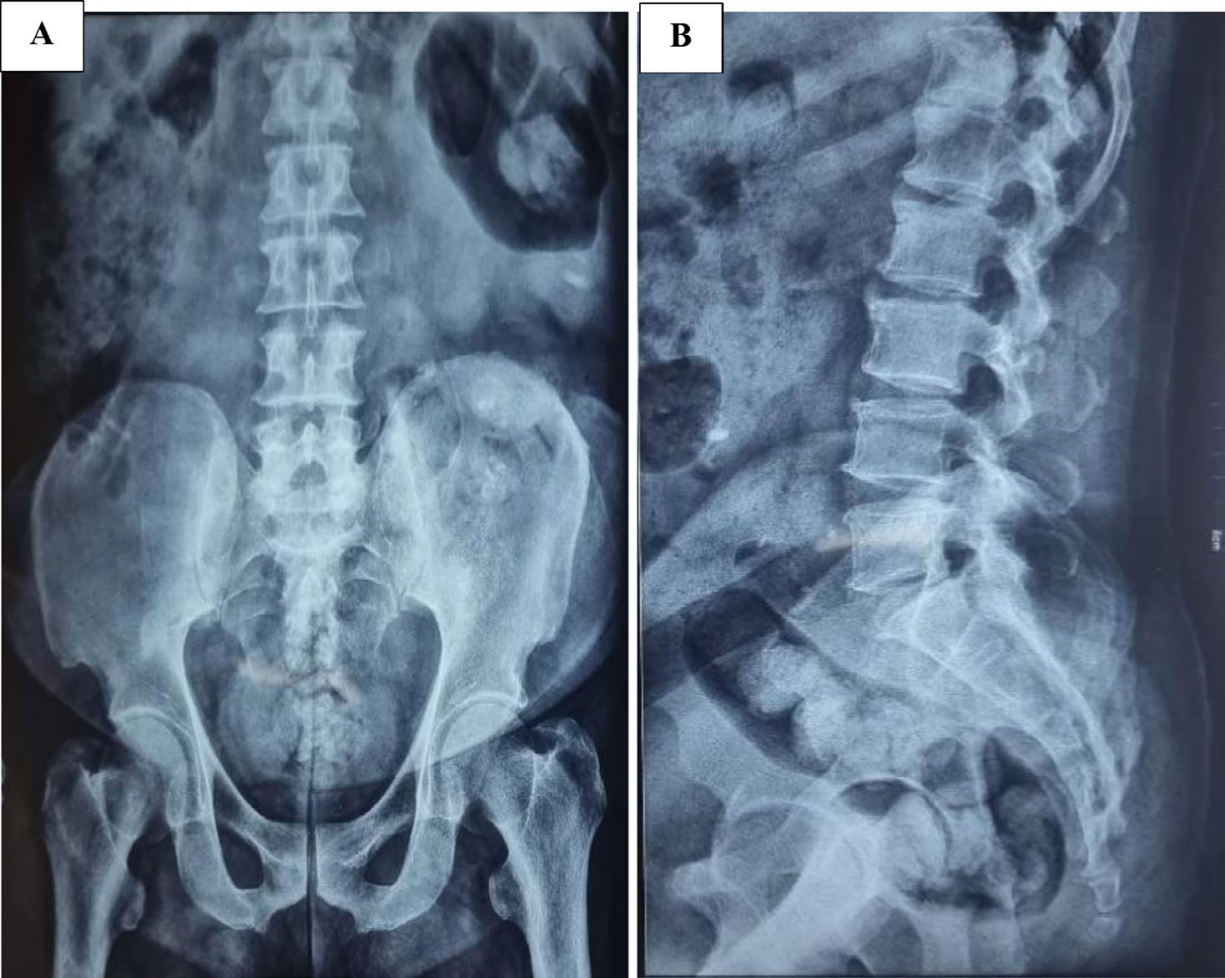
X‐ray abdomen anteroposterior view (A) and lateral view (B) showing distended bowel loops.

On an abdominal examination at our center, he had abdominal distention with absent bowel sounds. Neurological examination revealed a power of 0/5 on Medical Research Council grading with decreased sensation to touch and pain below the D10 spinal level. An X‐ray of the abdomen was done, which showed distended bowel loops (Figure 2).

**FIGURE 3:**
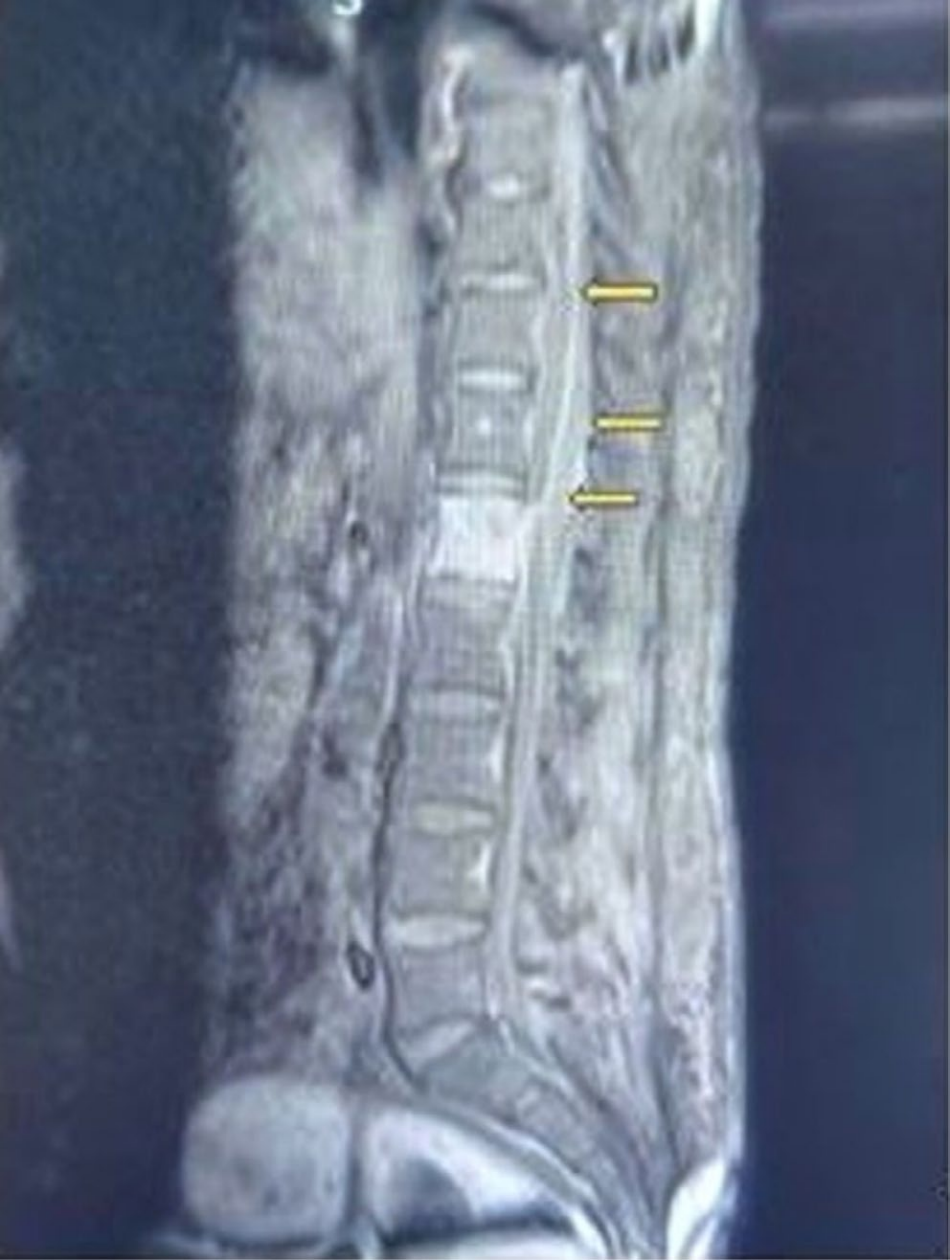
An MRI of the dorsolumbar spine showed an elongated area of peripherally enhancing altered signal intensity lesion in epidural space extending from D9 to L2 levels.

The patient improved his bowel symptoms from conservative treatment over the next 24 h. As the previous MRI failed to explain the current neurological deficit, a new MRI of the dorsolumbar spine was obtained. The scans showed a T1 low and T2/STIR high signal characteristics lesion in the D8 and D9 vertebrae and L1 vertebra, along with an elongated area of peripherally enhancing altered signal intensity lesion in the anterior epidural space extending from C7 to L1 vertebral level, with a similar lesion in the posterior epidural space from D4 to D6 and D12 to L1 levels. The spinal cord was extremely thinned out from D7 to D9 level. A diagnosis of an epidural abscess was made (Figure 3). The patient underwent an emergent surgery that involved a D10 laminectomy and drainage of purulent discharge with the help of a soft silicon catheter below and above the D10 level.

## 4 Conclusion and Results

The patient tolerated the surgery well and was eventually transferred to the neurosurgical ward on the 7th postoperative day. On microbiological examination, methicillin‐sensitive *Staphylococcus aureus* (MRSA) was isolated. *Mycobacterium tuberculosis* was not detected on the AFB stain or GeneXpert Ultra test. The venous doppler was negative for deep vein thrombosis (DVT). The patient was started on culture‐specific intravenous antibiotics, intensive physiotherapy, and DVT prophylaxis, while the ATT was discontinued. Despite a gradual improvement in the patient's condition, there was no change in the neurological status. An MRI was planned in 2 weeks. However, on day 18 of surgery, he suddenly developed dyspnoea and collapsed. Aggressive cardiopulmonary resuscitation was instituted that failed to revive the patient. As no autopsy was done, pulmonary embolism as a presumed cause of death was made.

## 5 Discussion

Early diagnosis and treatment of SEA is extremely important. Patients with SEA often present with non‐specific symptoms that include back pain, fever, motor weakness, sensory abnormalities, and bowel/bladder dysfunction [1, 2, 3]. A patient with SEA usually goes through four and often overlapping stages: spinal ache, root pain, weakness of voluntary muscles, sphincter, sensibilities, and paralysis (Table 1) [2, 3, 7, 8, 9, 10, 11]. As the patients often do not initially present with the more specific neurological symptoms, a delay in or a missed diagnosis can occur [2, 3, 12, 13]. Julio et al., in their study of 15 cases of SEA, found that the absence of neurologic signs and fever were the main reasons for delayed diagnosis [5]. Unfortunately, once the neurological manifestations set in, they generally indicate a worse prognosis for the patient's overall recovery [2, 12, 13, 14]. In our case, the patient was misdiagnosed with Pott's spine based on his initial symptom of low back pain, elevated inflammatory markers (ESR and CRP), and MRI findings. He was treated accordingly with ATT, but instead, he further developed bowel dysfunction along with neurological deficits. A repeat MRI helped to confirm the diagnosis of SEA.

**TABLE 1:** Stages of SEA according to the clinical symptoms' progression (adapted from Heusner et al. [7] and Peterson et al. [8]).

Stages	Symptoms
Stage 1	Aching pain at affected level of spine, fever, localized tenderness over spine
Stage 2	Nerve root pain, headache, neck stiffness, depressed deep tendon reflexes (DTRs) if lesion overlies cauda equina or heightening of DTRs if it is over spinal cord
Stage 3	Motor weakness, bowel and bladder dysfunction, sensory abnormalities such as ascending numbness
Stage 4	Complete paralysis

This case is unique as the patient presented with two acute conditions: long‐segment spinal epidural abscess and acute intestinal obstruction. An extensive search of the literature yielded several cases to date where spinal epidural abscess mimicked an abdominal pathology. Bremer et al. reported a case of spinal epidural abscess who initially presented with symptoms suggesting an intra‐abdominal pathology [9]. They also reported three previous similar reports of SEA mimicking acute appendicitis and cholecystitis [9, 13, 14, 15]. Four other cases have been reported where an abdominal pathology masked SEA (Table 2).

**TABLE 2:** Cases where spinal epidural abscess presented as an intra‐abdominal pathology.

Author	Age/sex	Risk factors present	Initial diagnosis
Bremer et al. [9]	62‐year‐old/male	Age, diabetes, hypertension, cervical osteomyelitis, psoriasis, degenerative changes in the thoracic vertebra	Post‐operative pancreatitis
Tyson et al. [13]	7‐year‐old/female	None reported	Appendicitis
Flikweert et al. [14]	7‐year‐old/male	None reported	Appendicitis
Lam and Hynes et al. [15]	65‐year‐old/male	Age	Cholecystitis
Cheng‐Chih et al. [16]	68‐year‐old/male	Age, hepatitis C	Gastric ulcer with degenerative spondylosis
Fakhouri F et al. [17]	13‐year‐old/female	None reported	Acute abdomen suspected complicated appendicitis

The SEA can present with abdominal symptoms such as bowel obstruction due to the complex interplay of neurological and inflammatory processes. The mechanism linking SEA to abdominal symptoms involves several factors: radicular pain and referred symptoms [13, 18], visceroparietal reflexes, spinal Ileus [13], and autonomic nervous system deficits [18, 19]. The coexistence of SEA with acute intestinal obstruction makes the management strategies complex. The symptoms of intestinal obstruction, which were more acute and dramatic, were addressed initially. This contributed to further delays in the diagnosis of the case.

Most of the patients with SEA tend to have multiple predisposing factors [3]. Diabetes mellitus, intravenous drug use, and substance abuse, including alcoholism, are the most common underlying conditions in SEA [1, 10, 11, 12, 20]. In the case of our patient, advancing age and diabetes mellitus could have been significant contributing factors to his clinical progression. The pus culture in our patient grew *S. aureus* , the most common organism isolated in SEA [1, 2, 3, 4, 5, 10, 12, 20]. Both the presence of diabetes mellitus and infection with *S. aureus* (methicillin‐resistant) are strong predictors of poor neurological outcomes in patients with SEA [20].

Gadolinium‐enhanced MRI remains the imaging method of choice [2, 3, 14, 16]. It is less invasive, defines the extension of the abscess, that is, longitudinal and paraspinal, and helps differentiate infection from cancer based on the appearance and signal intensity of the image [3]. The treatment of choice for neurologically symptomatic patients is urgent decompression followed by intravenous antibiotics. Patients with one or more underlying risk factors benefit from surgical intervention early in the disease course [1, 20]. Patel et al. reported significant impairment of motor function in patients who underwent surgery after a failed medical intervention. The baseline risk of failure rate of medical management is 8.3%, which increases significantly as more risk factors are present in the patient [12]. After an initial recovery period, our patient ultimately died. As an autopsy was not performed, pulmonary embolism remains the most probable cause of death due to the rapidity of the event. Vakili et al. report an improvement in mortality rates from 81% in the 1920s to 15% in 1991–1997, with lower rates reported in more recent studies, attributing it to earlier diagnosis and treatment of SEA. However, they agreed that further improvement in early diagnosis is still required as 15% of paralysis or deaths occurred in their review of cases from 2004 to 2014 at a large academic hospital [11].

SEA is an uncommon and potentially life‐threatening condition requiring early diagnosis and prompt treatment. Coexistence with acute intestinal obstruction is extremely rare. Delays in diagnosis and the presence of comorbidities can result in unfavorable outcomes, such as in our case.

## Author Contributions

**Shitashma Bohara:** conceptualization, data curation, formal analysis, investigation, methodology, project administration, resources, software, visualization, writing – original draft, writing – review and editing. **Susmin Karki:** conceptualization, data curation, formal analysis, investigation, methodology, project administration, resources, software, validation, visualization, writing – original draft, writing – review and editing. **Bikas Thapa:** conceptualization, resources, writing – original draft, writing – review and editing. **Niharika Khanal:** data curation, writing – original draft, writing – review and editing. **Bikal Ghimire:** supervision, writing – review and editing. **Sandeep Bohara:** supervision, writing – review and editing. **Amit Pradhanang:** writing – review and editing. **Mohan R. Sharma:** supervision, validation, visualization, writing – review and editing.

## Ethics Statement

The authors have nothing to report.

## Consent

Written informed consent was obtained from the patient to publish this report in accordance with the journal's patient consent policy.

## Conflicts of Interest

The authors declare no conflicts of interest.

## Data Availability

The data that support the findings of this study are available from the corresponding author, upon reasonable request.
